# Maintenance of Hokkaido virus, a genotype of *Orthohantavirus puumalaense*, in the rodent host *Myodes rufocanus bedfordiae* under natural conditions

**DOI:** 10.1128/jvi.00321-26

**Published:** 2026-06-30

**Authors:** Thi Ngoc Thuy Duong, Michihito Sasaki, Yasuko Orba, Keisuke Aoshima, Osamu Ichii, Keisuke Maezono, Passawat Thammahakin, Haruto Eguchi, Shintaro Kobayashi, Hiroaki Kariwa

**Affiliations:** 1 Laboratory of Public Health, Graduate School of Infectious Diseases, Hokkaido University 592521, Sapporo, Japan; 2 Tay Nguyen Institute of Hygiene and Epidemiology (TIHE) 613487 , Buon Ma Thuot, Dak Lak, Vietnam; 3 Division of Molecular Pathobiology, International Institute for Zoonosis Control, Hokkaido University 12810, Sapporo, Japan; 4 Institute for Vaccine Research and Development (HU-IVReD), Hokkaido University 12810, Sapporo, Japan; 5 International Collaboration Unit, International Institute for Zoonosis Control, Hokkaido University 12810, Sapporo, Japan; 6 Laboratory of Comparative Pathology, Faculty of Veterinary Medicine, Hokkaido University 12810, Sapporo, Japan; 7 One Health Research Center, Hokkaido University 12810, Sapporo, Japan; 8 Laboratory of Anatomy, Faculty of Veterinary Medicine, Hokkaido University 12810, Sapporo, Japan; 9 Veterinary Research Unit, International Institute for Zoonosis Control, Hokkaido University 12810, Sapporo, Japan; University of Freiburg, Freiburg, Germany

**Keywords:** *Orthohantavirus*, Hokkaido virus, *Myodes rufocanus bedfordiae*, natural infection, virus maintenance

## Abstract

A variety of orthohantaviruses (family *Hantaviridae*) have preferred natural host species, with transmission among hosts generally thought to occur through direct physical contact and inhalation of virus-contaminated excreta, although the infection to the other species occasionally occurs. Despite extensive experimental studies, the mechanisms of orthohantavirus maintenance and transmission under natural conditions remain unclear. In this study, field surveys were conducted in a forest in Tobetsu, Hokkaido, Japan, between 2022 and 2025 to capture gray red-backed voles (*Myodes rufocanus bedfordiae*), the natural host of Hokkaido virus (HOKV), a genotype of *Orthohantavirus puumalaense*. Among 199 captured rodents, 23 were positive for HOKV infection. Five individuals were positive for viral RNA but negative for anti-HOKV IgG antibodies on ELISA and IFA and exhibited low neutralizing antibody titers and low IgG avidity indexes (≤26%), suggesting acute infection. In contrast, 18 individuals were positive for viral RNA and showed high antibody titers on ELISA, IFA, and neutralization tests, as well as high IgG avidities (≥64%); these individuals were considered persistently infected. High levels of viral RNA and antigens were consistently detected in the lungs, kidneys, and spleen during both potential acute and persistent phases of HOKV infection by quantitative PCR and immunohistochemistry. Infectious HOKV was also recovered from oral swabs (8/8), urine (3/6), and feces (4/6) of individual rodents captured in 2024. These findings showed that HOKV can persist at high viral loads in host organs and be excreted throughout the course of infection, contributing to the long-term maintenance of orthohantavirus in natural host populations.

**IMPORTANCE:**

Diseases caused by zoonotic agents are major public health concerns. Orthohantaviruses are typical examples of zoonotic viruses transmitted from wild rodent hosts. Despite extensive laboratory investigations, the mechanisms of viral persistence and transmission in natural hosts remain poorly understood. Our study demonstrated that Hokkaido virus (HOKV), a genotype of *Orthohantavirus puumalaense*, seems to establish both acute and persistent infections in wild grey red-backed voles (*Myodes rufocanus bedfordiae*) without inducing major pathological changes. Detection of infectious virus in saliva, urine, and feces revealed multiple virus-shedding routes. Saliva likely serves as the predominant source of transmission, given that infectious virus was recovered from oral swabs of all infected rodents captured in 2024. These findings expand understanding of orthohantavirus ecology, persistence, and maintenance in reservoir populations. This information is crucial for evaluating spillover risks and enhancing public health preparedness.

## INTRODUCTION

Orthohantaviruses, members of the genus *Orthohantavirus* within the family *Hantaviridae*, are a diverse group of single-stranded, negative-sense RNA viruses. These viruses have a three-segmented RNA genome encoding the nucleocapsid (N) and, in some cases, nonstructural (NSs) proteins on the small (S) RNA segment, envelope glycoproteins (Gn and Gc) on the medium (M) RNA segment, and RNA-dependent RNA polymerase (RdRp) on the large (L) RNA segment (1, 2). Thirty-eight *Orthohantavirus* species have been identified to date, some of which are known to cause severe disease in humans and pose public health risks worldwide (1–3). Recent studies have identified a broader host range for hantaviruses, including shrews, moles, and bats (4–8). Host sharing, host-switching, and spillover events have occurred repeatedly during the evolutionary history of hantaviruses and have contributed to their complex evolutionary patterns and phylogeographic structure (8). Recently, it has been revealed that some orthohantavirus can infect multiple species of rodents (9, 10). Nevertheless, each orthohantavirus has a preferred host species and is considered to maintain a long-term reservoir host–virus relationship (11, 12). For example, *Orthohantavirus hantanense* (Hantaan virus, HTNV), *Orthohantavirus seoulense* (Seoul virus, SEOV), *Orthohantavirus puumalaense* (Puumala virus, PUUV), *Orthohantavirus andesense* (Andes virus, ANDV), and *Orthohantavirus sinnombreense* (Sin Nombre virus, SNV) are primarily carried by the striped field mouse (*Apodemus agrarius*), Norway rat (*Rattus norvegicus*), bank vole (*Myodes glareolus*), long-tailed pygmy rice rat (*Oligoryzomys longicaudatus*), and deer mouse (*Peromyscus maniculatus*), respectively (13–17).

Orthohantaviruses are believed to infect humans through inhalation of virus-containing excreta from infected animals or through animal bites. Human infections caused by orthohantaviruses are clinically classified into two main disease types: hemorrhagic fever with renal syndrome (HFRS), predominantly reported in Eurasia, and hantavirus pulmonary syndrome (HPS), occurring mainly in North and South America. Both diseases have high rates of morbidity and mortality (18, 19). In contrast to their pathogenicity in humans, previous studies have shown that orthohantaviruses typically persist without symptoms in their natural hosts, with detectable viral RNA in multiple tissues and viral maintenance lasting for over a year, even in the presence of a specific immune response (13, 20–24). Transmission among hosts is generally assumed to occur via direct physical contact, particularly biting involving virus-bearing saliva, or by inhalation of infectious aerosols generated from contaminated excreta (25–27). However, most data reported to date were derived from studies on experimentally infected animals, and limited information is available on the mode of orthohantavirus infection in individual rodents or the mechanisms of transmission among host populations in natural environments. Thus, field-based investigations are crucial for understanding how these viruses are maintained in their host populations over long periods, including aspects of virus tissue tropism, persistence in infected animals, and routes of excretion.

Hokkaido virus (HOKV) is a genotype of *O. puumalaense* (10, 28–30) and was first identified over 30 years ago in Tobetsu, Hokkaido, Japan, from a wild gray red-backed vole (*Myodes rufocanus bedfordiae*), a subspecies of *Myodes rufocanus* (31). *M. rufocanus* is geographically distributed across the northern Palearctic region, extending from northern Fennoscandia through Russia to northern Asia, including Hokkaido, Japan (32, 33). HOKV has been detected in several regions across this distribution range (31, 34–36). The HOKV lineages found in Hokkaido, Japan, and Sakhalin, Russia, are genetically close to PUUV strains hosted by *M. glareolus*. However, no human infections with HOKV have been reported to date, in contrast to PUUV, which has been shown to cause HFRS in Europe and Russia (10, 28, 29). Although *M. rufocanus bedfordiae* is a natural reservoir host of HOKV, little is known about the characteristics of HOKV-infected individuals and the ecology of HOKV within the rodent population. In this study, we conducted field surveys in Tobetsu, Hokkaido, Japan, from 2022 to 2025, and examined viral loads in various internal organs and excreta of naturally infected rodents. Our findings showed that orthohantaviruses can persist in host organs at high levels and be shed through excreta during both potentially acute and persistent phases of infection.

## MATERIALS AND METHODS

### Wild rodent sampling

A field investigation was conducted four times within a windbreak forest located in Tobetsu, Hokkaido, Japan, from 2022 to 2025 (43°12′21″ N, 141°25′18″ E), where HOKV is known to circulate among *M. rufocanus bedfordiae* populations. Wild rodents were captured using 100 Sherman live traps baited with oatmeal that were set overnight at each survey. A total of 199 rodents were captured and identified using a combination of morphological characteristics and nucleotide sequence analysis of the mitochondrial D-loop region (37). The rodent identification numbers were presented in the format of individual number/year. Under isoflurane anesthesia (Abbott Laboratories, Abbott Park, IL, USA), blood was collected via cardiac puncture. Urine, feces, and oral swab specimens were obtained, and internal organs (including the lungs, liver, spleen, kidneys, heart, salivary glands, and rectum) were collected and divided into two portions: one part was fixed in 10% neutral-buffered formalin (Wako Pure Chemicals, Osaka, Japan), and the other part was preserved in cryovials. All biological samples were immediately stored on ice and transported to the laboratory for further examination. Samples in formalin and cryovials were stored at room temperature (RT) and −80°C, respectively, until use. Sample preparation was performed in a biosafety level 3 containment laboratory, except for sample collections from animals that were carried out in the field.

### Serological screening for anti-orthohantavirus antibodies

Heat-inactivated (56°C, 30 min) serum samples from all captured rodents were screened for the presence of IgG antibodies against orthohantavirus antigens by indirect immunofluorescent antibody assay (IFA) and enzyme-linked immunosorbent assay (ELISA) as described previously (10, 29, 31).

### Indirect immunofluorescent antibody assay for detection of IgG (IgG-IFA) and IgM (IgM-IFA)

Monolayers of Vero E6 cells were infected with the PUUV strain Samara_94/CG/2009 (38) at MOI of 0.01 and incubated for 14 days. The infected cells were harvested by trypsinization and spotted onto 24-well slides (29, 39). After incubation for 4 h, the cells were fixed with methanol for 20 min, air-dried, and stored at −30°C until use as antigen slides. Rodent sera were diluted serially by two-fold (starting at 1:16) and tested for anti-hantavirus IgG antibodies using the antigen slides and Alexa Fluor 488-labeled protein G (1:500; Invitrogen). Monoclonal antibody E5/G6 was used as the positive control. For IgM detection, Alexa Fluor 488-conjugated anti-mouse IgM µ chain (1:500; Abcam, Cambridge, UK) was used. Sera from HOKV-infected hamsters were used as the positive controls. Scattered granular fluorescence in the cytoplasm was considered a positive reaction. IFA titers were determined as the reciprocal of the maximum dilution of serum that produced a positive signal.

### Enzyme-linked immunosorbent assay for IgG detection (IgG-ELISA)

Recombinant N protein (rNP) of HOKV was expressed as a fusion protein with the N-utilization substance A (NusA) protein, which was used as the specific antigen (31). The flat-bottom 96-well microtiter plates (Corning Inc., Corning, NY, USA) were coated overnight at 4°C with 50 μL per well of the rNP or the NusA diluted in 0.05 M carbonate/bicarbonate buffer (pH 9.8; Sigma) at a concentration of 1.6 μg/mL. The coated plates were blocked with a 1:5 dilution of Block Ace (Dai Nippon Pharmaceutical, Osaka, Japan) in deionized distilled water (DDW) and incubated at 37°C for 1 h. After washing three times with phosphate-buffered saline containing 0.05% Tween 20 (PBST), 2-fold serial dilutions from 1:100 to 1:6400 of serum samples were added to the plates (50 μL/well) and incubated at 37°C for 1 h. Each serum sample was reacted with the rN and the NusA protein. After the plates were washed three times with PBST, protein G–peroxidase (PO) conjugate (1:500; Zymed, San Francisco, CA, USA) was applied to detect antibodies (50 μL/well), and *o*-phenylenediamine (OPD) tablets (Sigma, St. Louis, MO, USA), together with hydrogen peroxide, served as the substrate for the peroxidase reaction (100 μL/well). The optical density at 450 nm (OD_450_) was measured using a plate reader (Multiskan MS; Labsystems Diagnostics Oy, Helsinki, Finland). The OD value of the rNP well minus that of the NusA well for the same serum was calculated and regarded as the ELISA value. ELISA titer was expressed as the reciprocal of the highest dilution showing an OD value of 0.2 or higher than 0.2. The cut-off value (0.2) was defined as the mean of negative sera plus three standard deviations (mean + 3 SD). Monoclonal antibody (mAb) E5/G6 to orthohantavirus N protein (40) was used as the positive control.

### IgG avidity assay by ELISA

IgG avidity was determined using a modified ELISA assay based on the previously described protocols (41–43). Briefly, diluted serum samples (1:100) were added to HOKV rNP- and NusA protein-coated wells and incubated under the same conditions as described for the ELISA assay. Each serum sample was tested in quadruplicate wells. Following incubation, one set of duplicate wells was washed three times sequentially with PBST, PBST containing 6 M urea, and PBST, whereas the corresponding duplicate wells were washed three times with PBST alone. After washing, the wells were incubated with protein A/G–peroxidase (PO) conjugate (Zymed, San Francisco, CA, USA), followed by the addition of the chromogenic substrate o-phenylenediamine (OPD) (Sigma, St. Louis, MO, USA). The optical density was measured at 450 nm (OD_450_). The OD value of the rNP well minus that of the NusA well with the same serum was calculated and regarded as the ELISA value. IgG avidity index was expressed as the percentage of the ELISA values obtained from the urea-washed wells to those from the PBST-washed wells.

### Molecular screening for viral RNA by RT-PCR

Lung tissues from all rodents were used for detection of viral RNA by reverse transcription polymerase chain reaction (RT-PCR). Total RNA from lungs was extracted using an ISOGEN II Kit (Nippon Gene, Tokyo, Japan). A partial sequence of the HOKV S-segment gene was amplified using the forward primer HOK_S_180Fw (5′-acggcaacaaacagtgtcag-3′), the reverse primer HOK_S_514Rv (5′-ggatcctcgtccctttgttt-3′), and the PrimeScript One-Step RT-PCR Kit (Takara Shuzo, Kyoto, Japan).

### Cell culture

MRK101 cells, a cell line derived from the kidney of *M. rufocanus bedfordiae* established previously (30), were maintained in Dulbecco’s modified Eagle’s medium (DMEM; high glucose; Wako Pure Chemicals) supplemented with 10% fetal bovine serum (FBS; Biowest, Nuaillé, France), 100 IU/mL penicillin, and 100 µg/mL streptomycin (both from Nacalai Tesque, Kyoto, Japan) at 37°C in a humidified atmosphere containing 5% CO_2_ and subcultured every 3–4 days.

### Virus titration

Infectious viral loads in oral swabs, urine, and feces from rodents or in culture supernatants after virus propagation were quantified using a focus-forming unit (FFU) assay, as described previously (10, 30). Samples were first subjected to serial dilution in DMEM and then inoculated onto confluent MRK101 cells cultured in 96-well plates. After adsorption at 37°C for 1 h, the inoculum was removed, and the cells were overlaid with DMEM supplemented with 1.5% carboxymethyl cellulose sodium salt (Wako Pure Chemicals) and 2% FBS. Infected monolayers were fixed 10 days post-infection with 100 µL of methanol per well for 15 min and then air-dried at RT. Viral foci were stained using the 2H10 mAb targeting the orthohantavirus nucleocapsid (N) protein (44), followed by Alexa Fluor 488-conjugated anti-mouse IgG (1:1000; Invitrogen, Carlsbad, CA, USA). Stained foci were counted under a fluorescence microscope. Oral swab, urine, and feces samples were diluted or suspended in phosphate-buffered saline (PBS), filtered through a 0.45 µm pore-size filter (Sartorius Stedim Biotech, Göttingen, Germany), and then used for virus titration assays.

### Focus reduction neutralization test

Neutralizing antibody titers were measured by focus reduction neutralization test (FRNT) as described previously (22, 31). Heat-inactivated rodent serum samples were serially diluted in DMEM. Equal volumes of diluted serum and virus stock solution (2.58 × 10^3^ FFU/mL) were mixed and incubated at 37°C for 1 h. Thereafter, the virus–serum mixture was inoculated onto a monolayer of MRK101 cells grown in 96-well plates (50 µL/well). After 1 h of incubation at 37°C, the inoculum was removed; cells were overlaid with DMEM containing 1.5% carboxymethyl cellulose sodium salt (Wako Pure Chemicals) and cultured in a humidified incubator at 37°C with 5% CO₂ for 10 days. Following incubation, the cells were washed and fixed with methanol. Viral foci were visualized with 50 µL of mAb E5/G6 (40), followed by Alexa Fluor 488-conjugated anti-mouse IgG (1:1000; Invitrogen). The FRNT titer of each serum sample was defined as the reciprocal of the highest serum dilution that produced at least an 80% reduction in foci number compared with the virus control.

### RNA extraction

Total RNA from tissues was extracted using an ISOGEN II Kit (Nippon Gene, Tokyo, Japan) in accordance with the manufacturer’s instructions. For oral swabs, urine, and feces, a High Pure Viral RNA Kit (Roche, Mannheim, Germany) was used. Approximately 50 mg of feces were homogenized in 500 µL of PBS, vortexed, and centrifuged at 4°C for 10 min at 4,000 rpm. Subsequently, 200 µL of the resulting supernatant was mixed with 400 µL of binding buffer containing linear polyacrylamide (5 ng/µL). Urine samples (50 µL) were diluted in 150 µL of PBS and then mixed with 400 µL of binding buffer containing linear polyacrylamide (5 ng/µL). Similarly, 200 µL aliquots of oral swab suspensions were directly combined with 400 µL of binding buffer containing linear polyacrylamide (5 ng/µL). All preparations were vortexed for 10 s, incubated at RT for 10 min, and processed via High Pure Filter Tubes (Roche), according to the manufacturer’s protocol. RNA was eluted in 50 µL of RNase-free water, quantified with a NanoDrop One Microvolume Spectrophotometer (Thermo Scientific, Waltham, MA, USA), and stored at –80°C until analysis.

### cDNA synthesis and qPCR analysis

First-strand cDNA was synthesized from total RNA using a PrimeScript II First-Strand cDNA Synthesis Kit (Takara Shuzo, Kyoto, Japan) according to the manufacturer’s instructions. An aliquot of 1 μL of the synthesized cDNA served as the template for qPCR using a KAPA Probe Fast qPCR Master Mix (2×) Kit (Nippon Gene), and each sample was tested in triplicate using primers and a probe targeting the S segment of HOKV as described previously (10): forward primer HOKV_S_225Fw (5′-caagcgaagaatggcagatgt-3′), reverse primer HOKV_S_343Rv (5′-cattcccatatctgaggcta-3′), and FAM-labeled probe HOKV_S_274 (FAM–5′-cctgctgacccgactg-3′–MGB). Thermal cycling was performed on a 7500 Fast Real-Time PCR System v2.0.6 (Applied Biosystems, Foster City, CA, USA) with an initial denaturation at 95°C for 3 min, followed by 40 cycles of 95°C for 3 s and 60°C for 30 s. Copy numbers of the HOKV S segment were determined using the standard curve method, and results were expressed as viral RNA copies per microgram of total RNA.

### Histopathology and immunohistochemistry

Formalin-fixed tissues were subjected to standard processing, including trimming, alcohol dehydration, and embedding in paraffin. Sections 4 µm thick were cut from each tissue block, mounted on adhesive glass slides (Matsunami Glass, Kishiwada, Japan), and stained with hematoxylin and eosin (H&E) for histopathological examination. For immunohistochemistry (IHC), deparaffinized sections were subjected to antigen retrieval using citrate buffer (10 mM, pH 6.0) in a pressure cooker, followed by peroxidase blocking. Primary antibody incubation was performed using the E5/G6 mouse monoclonal anti-orthohantavirus N protein antibody (40) at a 1:500 dilution in 1% bovine serum albumin (BSA; Sigma) in PBS overnight at 4°C under humidified conditions. Immunostaining was developed using a Histofine Simple Stain MAX PO (M) Kit and Histofine DAB Substrate Kit (Nichirei Bioscience, Tokyo, Japan), according to the manufacturer’s instructions. Slides were then counterstained with hematoxylin, dehydrated, cleared in xylene, and mounted with coverslips. Viral antigen signals were visualized under a light microscope (BX60; Olympus, Tokyo, Japan).

### Immunofluorescence staining for cellular markers

Tissue slides were prepared and subjected to antigen retrieval as described for IHC. The slides were then blocked with 10% normal goat serum (Nichirei Bioscience) for 10 min at RT, followed by overnight incubation at 4°C with primary antibodies diluted in 1% BSA in PBS. Double immunofluorescence staining was performed to assess viral tropism using mAb E5/G6 (1:500) for viral antigen detection combined with cell-specific markers: rabbit anti-CD34 (1:200; Proteintech, Rosemont, IL, USA) for capillary endothelium, rabbit anti-podoplanin (1:250; Proteintech) for type I pneumocytes, rabbit anti-surfactant protein C (SFTPC) (1:250; Proteintech) for type II pneumocytes, and rabbit anti-ionized calcium-binding adaptor molecule 1 (Iba-1) (1:500; Wako Pure Chemicals) for macrophages. Secondary antibodies included Alexa Fluor 488-conjugated anti-mouse IgG and Alexa Fluor 555-conjugated anti-rabbit IgG (1:500; Invitrogen). Nuclear counterstaining was performed with Hoechst 33342 (Invitrogen). Fluorescence images were acquired using a Zeiss LSM700 confocal laser scanning microscope with ZEN software (Zeiss, Oberkochen, Germany). Color optimization, quantitative image analysis, and cell counting were performed using Fiji software (an ImageJ distribution; NIH, Bethesda, MD, USA). For confocal analysis, at least five randomly selected fields per tissue section were analyzed to determine the number of viral antigen-positive cells with or without co-staining of the cell-type marker. Data are presented as the mean ± standard error of the mean (SEM) from tested animals (*n* = 4 to 5). Differences between two independent groups were analyzed using the Mann–Whitney U test. The statistical significance was established a priori at *P* < 0.05.

### Transmission electron microscopy

MRK101 cells were infected with HOKV strain Tobetsu_29/2022 (10) or PUUV strain Samara_94/CG/2005 (38) at a multiplicity of infection of 0.01. The infected cells were cultured in DMEM supplemented with 2% FBS for 7 days at 37°C in a CO_2_ incubator. The cells were then trypsinized and collected by centrifugation at 1200 rpm for 5 min. The resulting pellets were suspended in 2% agarose in PBS maintained at approximately 50°C. After the agarose had solidified, the gel containing infected cells was fixed in 1 mL of a mixture of 2.5% glutaraldehyde and 2% paraformaldehyde in 0.1 M cacodylate buffer for 2 h. The fixative was discarded, and the cells were washed four times with 1 mL of 0.1 M cacodylate buffer at 4°C. The samples were then fixed with 1% osmium tetroxide in 0.1 M cacodylate buffer at RT for 1 h and washed four times with 0.5 mL of water. Samples were stained with 1% uranyl acetate at 4°C overnight, washed four times with water, and dehydrated through a graded ethanol series (30%, 50%, 70%, 80%, 90%, 95%, and 100%). The ethanol was replaced with QY-1, and the samples were embedded in Epon resin (TAAB Laboratories Equipment Ltd., Reading, UK). The resin was solidified at 60°C for 2 days, sectioned into ultrathin slices using a diamond knife, and collected onto grid meshes. The samples on the grids were stained with 0.5% uranyl acetate in 50% methanol for 5 min at RT, followed by staining with 0.4% lead citrate for 3 min at RT. The prepared samples were examined by transmission electron microscopy (TEM) (JEM-1400Plus; JEOL, Tokyo, Japan). Formalin-fixed lung specimens from HOKV-infected and noninfected *M. rufocanus bedfordiae* were cut into 1 mm^3^ cubes and fixed in a mixture of 2.5% glutaraldehyde and 2% paraformaldehyde. Rodent lung samples were then processed according to the same protocol used for electron microscopy of cultured cells.

## RESULTS

### Rodent survey and laboratory screening

A total of 199 rodents were captured during four field surveys conducted in the forest of Tobetsu Town, Hokkaido Prefecture, Japan, from 2022 to 2025 (Table 1). Of these, 81.9% (163/199) were identified as *M. rufocanus bedfordiae*, 13.6% (27/199) as *Apodemus speciosus,* and 4.5% (9/199) as *Apodemus argenteus*. All collected specimens were examined for antibodies to HOKV in serum samples and for viral RNA in lung tissues. Twenty-three of the 199 rodents (11.6%) tested were positive for HOKV infection, as determined by antibody and/or viral RNA detection: 13.5% (22/163) in *M. rufocanus bedfordiae* and 3.7% (1/27) in *A. speciosus*. Among these, 18 individuals were seropositive by IgG-ELISA and IgG-IFA and also harbored viral RNA, whereas five individuals (#4/2022, #5/2024, #28/2024, #13/2025, and #14/2025) were negative for IgG by both ELISA and IFA but positive for viral RNA. Anti-HOKV IgM antibodies were detected at low IFA titers (ranging from 16 to 64) in any of 23 RNA-positive rodents (Tables 1 and 2).

**TABLE 1:** Prevalence of anti-HOKV antibodies and viral RNA among wild rodents captured in Tobetsu in surveys conducted from 2022 to 2025

Date of survey	Prevalence (%)							
	Antibody^*a*^				Viral RNA^*b*^			
	*M. rufocanus bedfordiae*	*Apodemus speciosus*	*Apodemus argenteus*	Total	*M. rufocanus bedfordiae*	*Apodemus speciosus*	*Apodemus argenteus*	Total
Aug 2022	3/42 (7.1)	1/2 (50.0)	0/0 (0.0)	4/44 (9.1)	4/42 (9.5)	1/2 (50.0)	0/0 (0.0)	5/44 (11.4)
Sep 2023	2/44 (4.5)	0/9 (0.0)	0/3 (0.0)	2/56 (3.6)	2/44 (4.5)	0/9 (0.0)	0/3 (0.0)	2/56 (3.6)
Sep 2024	6/34 (17.6)	0/5 (0.0)	0/0 (0.0)	6/39 (15.4)	8/34 (23.5)	0/5 (0.0)	0/0 (0.0)	8/39 (20.5)
Sep 2025	6/43 (14.0)	0/11 (0.0)	0/6 (0.0)	6/60 (10.0)	8/43 (18.6)	0/11 (0.0)	0/6 (0.0)	8/60 (13.3)
Total	17/163 (10.4)	1/27 (3.7)	0/9 (0.0)	18/199 (9.0)	22/163 (13.5)	1/27 (3.7)	0/9 (0.0)	23/199 (11.6)

^*a*^ Antibody to HOKV was tested by IgG-ELISA and IgG-IFA.

^*b*^ RT-PCR was used to detect viral RNA targeting the S-segment.

**TABLE 2:** Screening of anti-hantavirus antibodies, neutralizing antibodies against HOKV strain Tobetsu 29M/2022, and viral RNA in captured rodents from 2022 to 2025 (*N* = 199)^*f*^

Survey year	Total sample	Rodent no.	Sex	Species	IgM- IFA (titer)^*a*^	IgG- IFA (titer)^*a*^	IgG- ELISA (OD_1:100)^*b*^	IgG-ELISA (titer)^*c*^	IgG avidity (%)^*d*^	Neutralizing antibody titer (FRNT_80_)^*e*^	RT-PCR (viral RNA)
2022 (*N* = 44)	5	**4**	**F**	***M. rufocanus bedfordiae***	**+** (**32**)	**−** (**<16**)	**−** (**0.057**)	**−** (**<100**)	**15**	**80**	**+**
		9	F	*Apodemus speciosus*	+ (64)	+ (32)	+ (0.837)	+ (100)	66	320	**+**
		25	F	*M. rufocanus bedfordiae*	+ (32)	+ (64)	+ (2.339)	+ (400)	70	640	**+**
		29	F	*M. rufocanus bedfordiae*	+ (16)	+ (128)	+ (1.705)	+ (100)	78	1,280	**+**
		38	M	*M. rufocanus bedfordiae*	+ (32)	+ (256)	+ (1.482)	+ (100)	64	320	**+**
	39				NT	**−**	**−**	NT	NT	NT	**−**
2023 (*N* = 56)	2	37	F	*M. rufocanus bedfordiae*	+ (16)	+ (128)	+ (0.914)	+ (100)	70	640	**+**
		48	F	*M. rufocanus bedfordiae*	+ (32)	+ (64)	+ (1.899)	+ (200)	98	>2,560	**+**
	54				NT	**−**	**−**	NT	NT	NT	**−**
2024 (*N* = 39)	8	**5**	**M**	***M. rufocanus bedfordiae***	**+** (**32**)	**−** (**<16**)	**−** (**0.067**)	**−** (**<100**)	**25**	**160**	**+**
		**28**	**M**	***M. rufocanus bedfordiae***	**+** (**16**)	**−** (**<16**)	**−** (**0.017**)	**−** (**<100**)	**24**	**320**	**+**
		11	F	*M. rufocanus bedfordiae*	+ (16)	+ (32)	+ (2.627)	+ (800)	73	320	+
		16	M	*M. rufocanus bedfordiae*	+ (32)	+ (128)	+ (1.626)	+ (100)	89	1,280	+
		20	M	*M. rufocanus bedfordiae*	+ (32)	+ (32)	+ (2.778)	+ (800)	96	>2,560	+
		25	M	*M. rufocanus bedfordiae*	+ (16)	+ (256)	+ (2.454)	+ (200)	98	>2,560	+
		27	F	*M. rufocanus bedfordiae*	+ (16)	+ (128)	+ (0.415)	+ (100)	68	640	+
		33	M	*M. rufocanus bedfordiae*	+ (16)	+ (64)	+ (2.362)	+ (400)	93	>2,560	+
	31				NT	**−**	**−**	NT	NT	NT	**−**
2025 (*N* = 60)	8	**13**	**M**	***M. rufocanus bedfordiae***	**+** (**32**)	**−** (**<16**)	**−** (**0.096**)	**−** (**<100**)	**26**	**160**	**+**
		**14**	**M**	***M. rufocanus bedfordiae***	**+** (**32**)	**−** (**<16**)	**−** (**−0.023**)	**−** (**<100**)	**12**	**80**	**+**
		18	M	*M. rufocanus bedfordiae*	+ (32)	+ (512)	+ (1.939)	+ (200)	74	640	+
		24	F	*M. rufocanus bedfordiae*	+ (16)	+ (1,024)	+ (1.904)	+ (100)	93	1,280	+
		37	F	*M. rufocanus bedfordiae*	+ (32)	+ (128)	+ (0.669)	+ (100)	78	320	+
		53	F	*M. rufocanus bedfordiae*	+ (16)	+ (512)	+ (2.012)	+ (200)	90	1,280	+
		58	M	*M. rufocanus bedfordiae*	+ (32)	+ (256)	+ (1.773)	+ (200)	80	640	+
		59	F	*M. rufocanus bedfordiae*	+ (16)	+ (1,024)	+ (2.655)	+ (800)	93	>2,560	+
	52				NT	**−**	**−**	NT	NT	NT	**−**

^*a*^ IFA antibody titer was expressed as the reciprocal of the highest dilution showing the specific granules in the cytoplasma.

^*b*^ OD value was measured at a serum dilution of 1:100. Cut off value = 0.2, defined as the mean of negative sera plus three standard deviations (mean + 3 SD).

^*c*^ ELISA titer was expressed as the reciprocal of the highest dilution showing OD value 0.2 or higher.

^*d*^ IgG avidity index was calculated as the percent of ELISA value by urea wash to that by PBST wash only.

^*e*^ FRNT_80_ value was expressed as the reciprocal of the highest dilution showing 80% or higher focus reduction.

^*f*^ E5/G6 monoclonal antibody targeting the orthohantavirus nucleocapsid protein used as a positive control for IgG-ELISA and IgG-IFA. F, female; M, male; −, negative; +, positive; NT, not tested. Bold characters indicate rodents that may have been in the acute phase of HOKV infection.

To further characterize the infection status of HOKV-infected rodents, focus reduction neutralization tests and IgG avidity assays were performed. The five IgG-ELISA- and IgG-IFA-negative but RNA-positive rodents showed low neutralizing antibody titers (≤320) and low IgG avidity indexes (≤26%), whereas ELISA- and IFA-positive rodents exhibited higher neutralizing antibody titers ranging from 320 to >2,560, together with high IgG avidity indexes ranging from 64% to 98% (Table 2). These findings suggest that individuals #4/2022, #5/2024, #28/2024, #13/2025, and #14/2025 were potentially in the acute phase of infection, while the remaining 18 positive rodents were likely in the persistent phase of infection.

### Quantification of viral RNA in organs of naturally infected rodents

**Fig 1:**
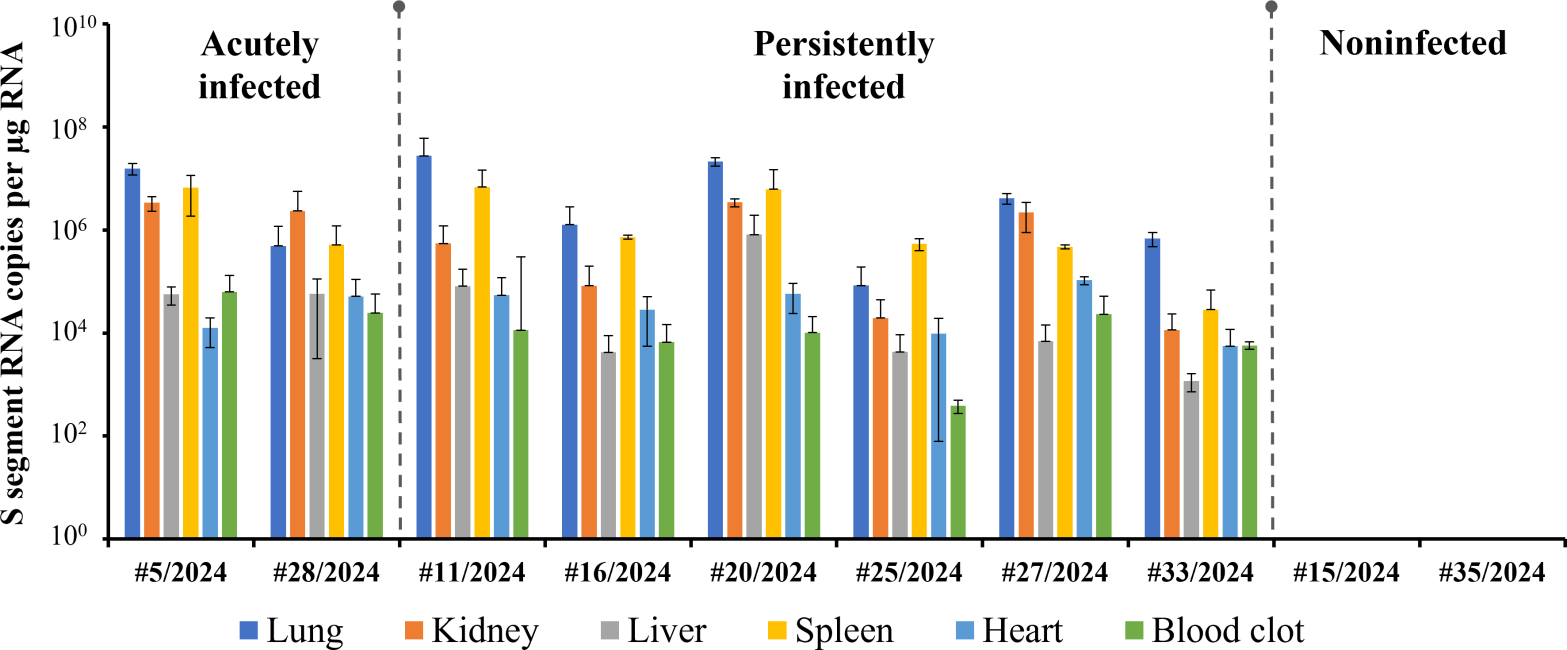
Demonstration of HOKV viral loads in tested organs and blood samples of naturally infected *M. rufocanus bedfordiae* captured in a forest in Tobetsu, Hokkaido, Japan. Each organ was tested by qPCR to quantify HOKV S-segment copy numbers (expressed as copies per microgram of total RNA). Rodents #5/2024, #11/2024, #16/2024, #20/2024, #25/2024, #27/2024, #28/2024, and #33/2024 were positive for HOKV infection. Rodents #15/2024 and #35/2024 were included as HOKV-negative controls. Error bars represent standard error of the mean.

Total RNA was extracted from blood clots and major organs, including the lungs, kidneys, liver, spleen, heart, salivary gland, and rectum, of 23 HOKV-infected rodents and five noninfected rodents (#15/2024, #24/2024, #35/2024, #43/2025, and #50/2025). qPCR targeting the viral S segment was performed to determine viral RNA loads (Fig. 1; Table S1). HOKV RNA was detected in multiple organs of all infected rodents, at levels ranging from 4.6 × 10^1^ to 2.9 × 10^7^ copies per microgram of total RNA, with distribution varying among organs and individuals. In all infected rodents, high viral RNA loads were observed in the lungs, kidneys, and spleen. In addition, viral RNA was consistently detected in blood clots from all RNA-positive animals, suggesting viremia during both acute and persistent phases of infection. No differences in viral load patterns across organs were observed between rodents in potential acute and persistent phases. HOKV RNA was undetectable in tissues and blood from noninfected rodents (Fig. 1; Table S1).

### Detection of HOKV antigens in wild rodent organs

**Fig 2:**
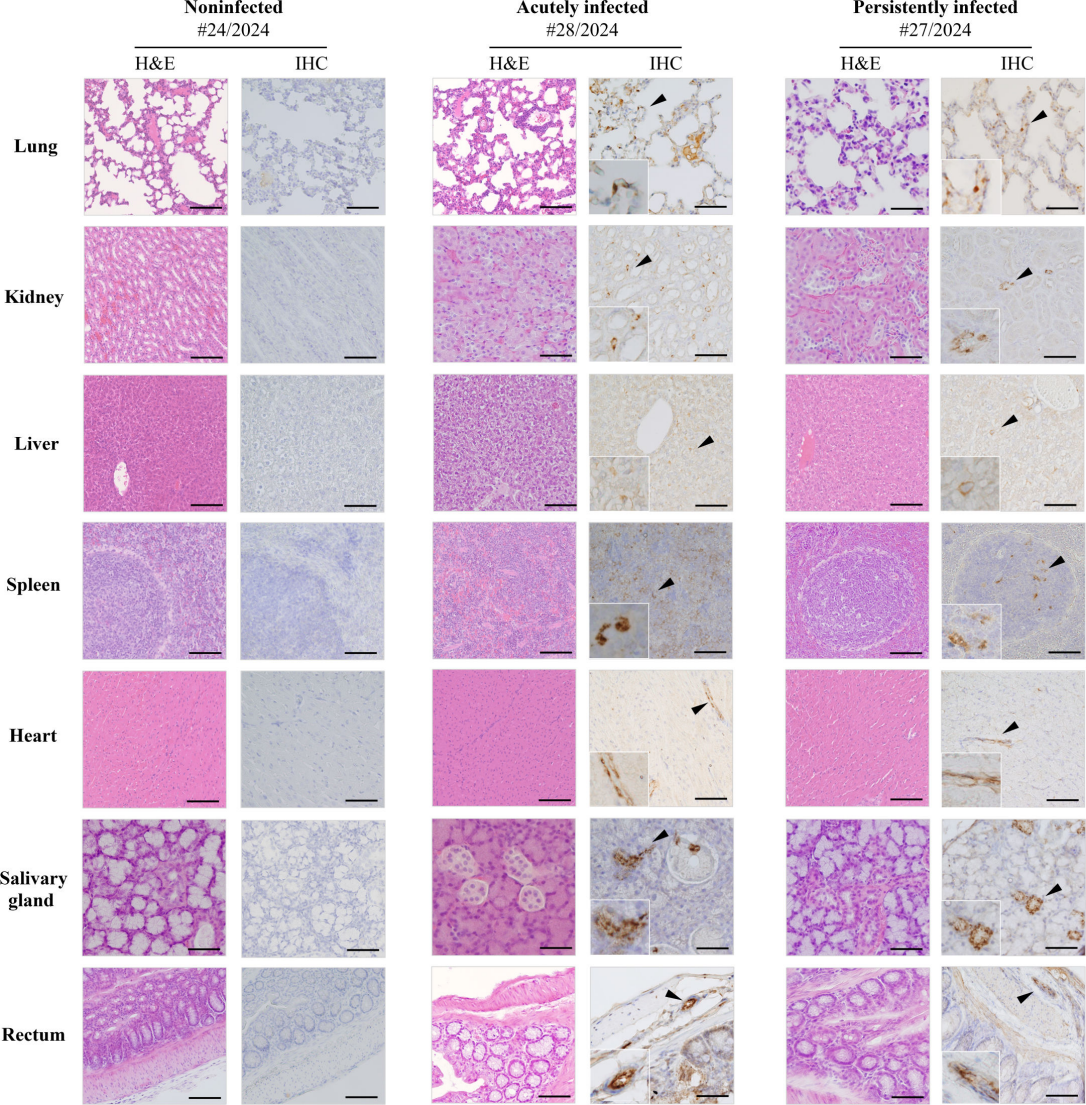
Evidence of orthohantavirus antigens in organs of wild *M. rufocanus bedfordiae*. Pathological and immunohistochemical examination of the lungs, kidneys, liver, spleen, heart, salivary glands, and rectum of naturally infected rodents. Representative images are shown for organ sections from an animal per group, including potential acute infection (#28/2024), potential persistent infection (#27/2024), and noninfection (#24/2024), stained with hematoxylin and eosin (H&E; scale bars, 100 µm) or by immunohistochemistry (IHC) for orthohantavirus nucleoprotein (N) (scale bars: 100 µm). Left inset images show higher magnification of IHC-positive cells indicated by arrowheads.

To determine whether HOKV causes pathological changes in the host and whether viral antigens persist in the organs of natural hosts, tissue samples from the lungs, kidneys, liver, spleen, heart, salivary glands, and rectum of four rodents in potential acute phase (#5/2024, #28/2024, #13/2025, and #14/2025), four rodents in potential persistent phase (#25/2024, #27/2024, #37/2025, and #53/2025), and four noninfected rodents (#15/2024, #24/2024, #43/2025, and #50/2025) were examined histopathologically by H&E staining and analyzed by IHC (Fig. 2; Fig. S1). Histopathological examination of lung sections revealed mild inflammatory cellular infiltration into the pulmonary interstitium accompanied by thickening of the alveolar walls in some infected individuals (rodent in potential acute phase #13/2025 and rodent in potential persistent phase #37/2025). However, similar pathological changes were also observed in noninfected rodent #15/2024, which exhibited marked alveolar edema (Fig. S1). IHC analysis demonstrated positive antigen signals predominantly localized in the alveolar regions of infected rodents, particularly within the vascular endothelium and alveolar septa, whereas no such signals were detected in noninfected animals (Fig. 2). These findings suggest that the presence of viral antigen in the lung is not a definitive indicator of pathological changes. Kidney sections from the examined rodents showed no apparent histopathological abnormalities. However, IHC analysis detected viral antigens localized within the renal endothelium, including the glomeruli and intertubular capillaries, in infected individuals (Fig. 2; Fig. S1). In both infected and noninfected animals, pathological changes were observed in the liver and spleen, possibly attributable to bacterial or parasitic infection, with splenomegaly and extramedullary hematopoiesis noted in the spleen (Fig. S1). Consistent with the results of qPCR, IHC analysis revealed the presence of HOKV antigens in multiple organs of infected rodents, with the highest frequency of positive signals in the lungs, kidneys, and spleen. Moderate immunopositivity was observed in the heart, salivary glands, and rectum, whereas only sparse immunostaining was detected in the liver (Fig. 2; Fig. S1). The observations were essentially similar in both phases of infection; no obvious histopathological lesions were identified in any of the examined organs that could be convincingly associated with HOKV infection.

**Fig 3:**
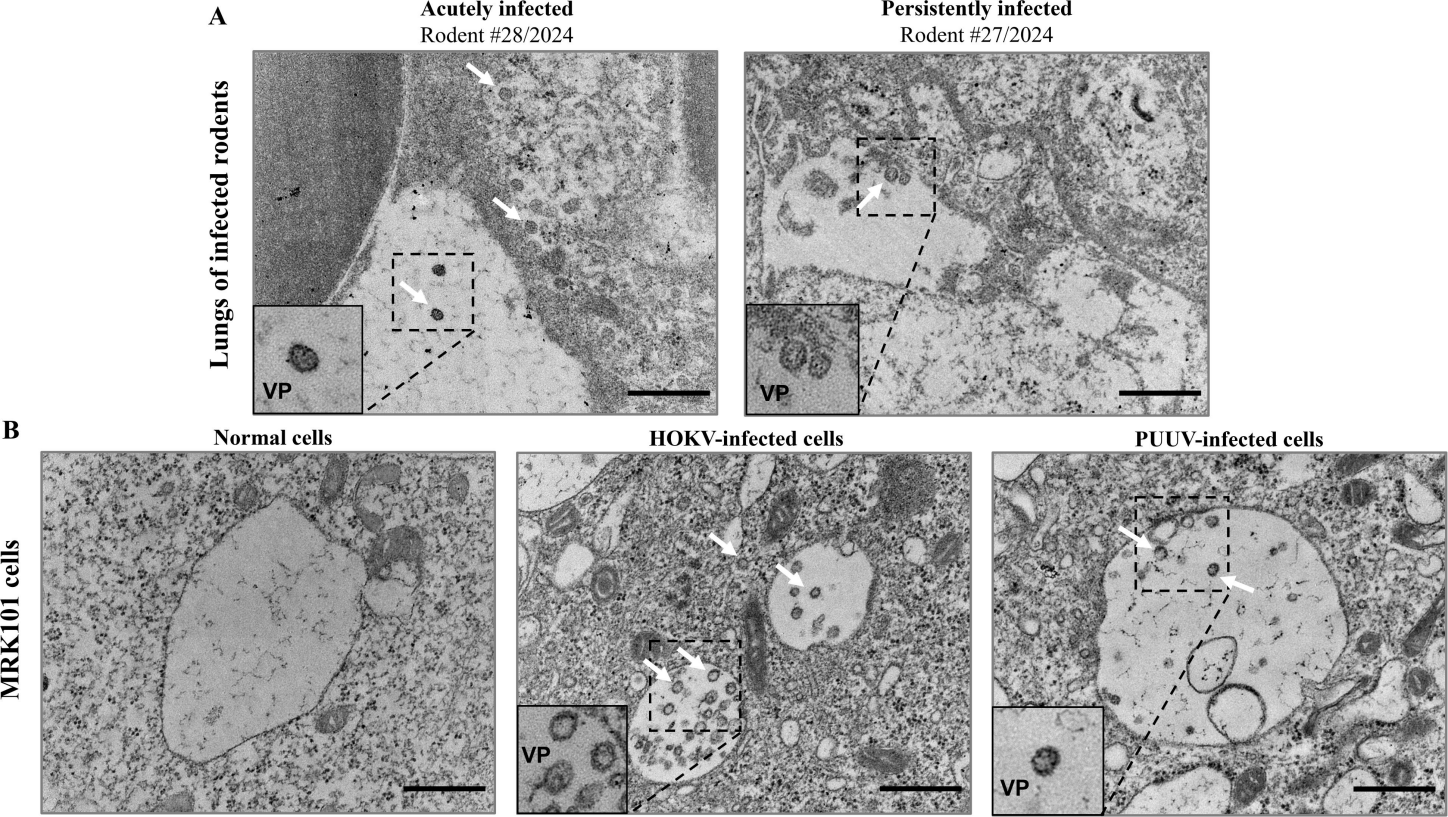
Morphological characterization of orthohantavirus-like particles in tissues and cells. (**A**) Electron microscopy of lung specimens from representative *M. rufocanus bedfordiae* with potential acute infection (#28/2024) and potential persistent infection (#27/2024). (**B**) Thin sections showing a general view of MRK101 cells infected with HOKV strain Tobetsu_29M/2022 or PUUV strain Samara_94/CG/2009 (7 days post-infection), along with uninfected MRK101 cells, were analyzed by transmission electron microscopy (TEM). Arrows indicate virus particles. Left inset: higher magnification of the area outlined by the square, showing virus-like particles (VPs). Scale bar: 500 nm.

Formalin-fixed lung specimens from HOKV-infected *M. rufocanus bedfordiae* were processed for TEM to confirm the presence and examine the ultrastructural morphology of orthohantavirus particles in naturally infected rodents (Fig. 3A). Ultrathin sections of MRK101 cells infected with either HOKV strain Tobetsu_29M/2022 or PUUV strain Samara_94/CG/2009, along with uninfected control cells, were also examined by TEM to verify the morphological characteristics of orthohantavirus-like particles (Fig. 3B). TEM analysis revealed orthohantavirus-like particles with diameters ranging from 75 to 140 nm in lung sections from wild rodents in both potential acute and persistent infection groups. The morphologies of these viral particles resembled those observed in orthohantavirus-infected MRK101 cells.

### HOKV cell tropism in the host lung and kidney

**Fig 4:**
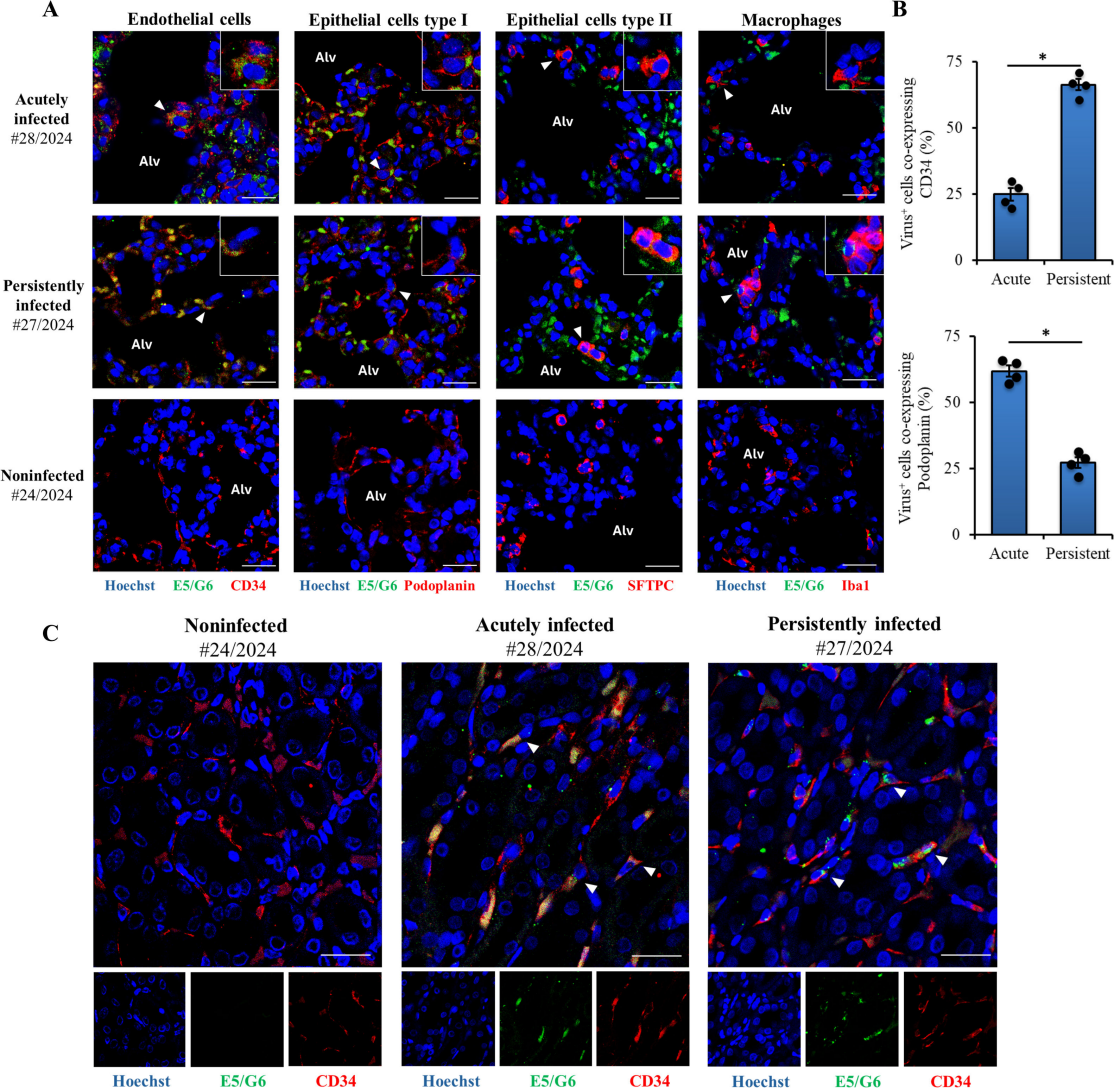
Orthohantavirus cell tropism in the lung and kidney of wild rodents. As representative examples, lung and kidney sections from *M. rufocanus bedfordiae* with potential acute infection (#28/2024), potential persistent infection (#27/2024), and a noninfected control (#24/2024) were examined by immunofluorescence staining. (**A**) Lung sections were immunostained with antibodies for orthohantavirus N (green) and cell-type markers CD34, podoplanin, SFTPC, and Iba-1 for microvascular endothelial cells, type I alveolar epithelial cells, type II alveolar epithelial cells, and macrophages, respectively (red). Cell nuclei were visualized with Hoechst 33342 staining (blue). Top right inset images show higher magnification views of individually infected cells, indicated by arrowheads. Alv, alveolar lumen. Scale bars: 20 μm. (**B**) The percentage of orthohantavirus N protein–positive cells co-stained with a cell-type marker (CD34 for microvascular endothelial cells or podoplanin for type I epithelial cells) was quantified. Data are presented as the mean ± SEM from four animals per group (*n* = 4). Statistical comparisons between the acutely and persistently infected groups were performed using a two-tailed Mann–Whitney U test. *P < 0.05. (**C**) Kidney sections were immunostained with antibodies for orthohantavirus antigen (green) and CD34 as a marker of microvascular endothelial cells (red). Nuclei are shown in blue. Representative colocalization of endothelial cell signals and viral antigen signals is indicated by arrowheads. Scale bars: 20 μm.

To identify cell types in the lung and kidney susceptible to HOKV infection, organ sections from 4 *M. rufocanus bedfordiae* in each group—potential acute infection (#5/2024, #28/2024, #13/2025, and #14/2025), potential persistent infection (#20/2024, #25/2024, #27/2024, and #53/2025), and noninfection (#24/2024, #35/2024, #43/2025, and #50/2025)—were subjected to immunofluorescence co-staining using antibodies against the orthohantavirus antigen (E5/G6) in combination with cell-type markers, including CD34 for microvascular endothelial cells, podoplanin for type I alveolar epithelial cells, SFTPC for type II alveolar epithelial cells, and Iba-1 for macrophages (Fig. 4; Fig. S2 and S3). In the lung, viral antigens were predominantly localized to the alveolar septa. During the potential acute infection phase, antigen signals were primarily co-localized with podoplanin^+^ type I alveolar epithelial cells, whereas in the potential persistent phase, they were more frequently co-localized with CD34^+^ microvascular endothelial cells (Fig. 4A and B). In both potential acute and persistent infections, occasional viral antigen localization was detected in type II alveolar epithelial cells, and limited localization was observed in macrophages. The images of three representative individuals from each group were presented in Fig. 4A and Fig. S2. In the kidney, immunofluorescence analysis revealed that viral antigens were mainly restricted to CD34^+^ endothelial cells; this distribution pattern was consistent across both potential acute and persistent phases of infection in wild rodents (Fig. 4C; Fig. S3). In summary, HOKV may exhibit different cell tropism patterns in *M. rufocanus bedfordiae*. In the lung, viral antigens showed preferential localization to type I alveolar epithelial cells during the potential acute phase, whereas endothelial localization predominated during the potential persistent phase. In contrast, viral antigens were predominantly localized in endothelial cells in the kidney throughout the course of infection.

### Viral shedding patterns of HOKV-infected individual rodents

**Fig 5:**
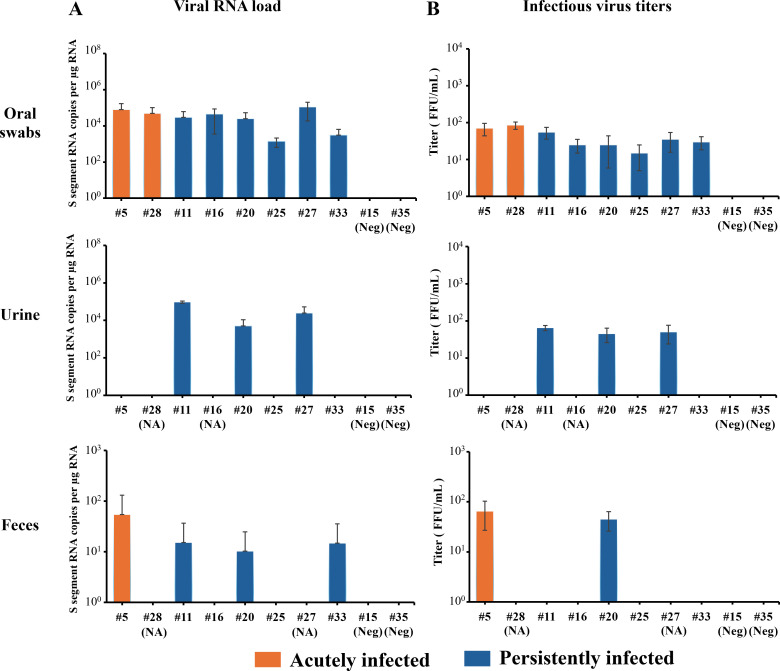
Shedding of HOKV in wild rodents captured in 2024. (**A**) Viral RNA copy numbers in oral swabs, urine, and feces of naturally infected *M. rufocanus bedfordiae* assessed by qPCR. (**B**) Oral swab, urine, and fecal samples were suspended or diluted in PBS and filtered through a 0.45 µm membrane filter. The infectious virus titer of each sample was determined by focus-forming unit assay (FFU/mL). Data are shown as mean ± SEM; NA, not available.

To identify the sources of infection contributing to HOKV transmission among rodents in the wild, RNA loads and infectious titers of HOKV in oral swabs, urine, and feces from eight HOKV-positive rodents captured in 2024 were quantified by qPCR and FFU assays (Fig. 5A and B). HOKV RNA was consistently detected in oral swabs from all eight infected rodents, with viral RNA loads ranging from 10^3^ to 10^5^ copies per microgram of total RNA. There were no significant differences in viral RNA copy numbers in oral swabs between rodents in potential acute and persistent infection phases (Fig. 5A). Infectious virus was also recovered from the oral swabs of all eight infected animals (Fig. 5B). In contrast, urinary excretion of both viral RNA and infectious virus was detected exclusively in persistently infected individuals #11/2024, #20/2024, and #27/2024, but was undetectable in the other infected rodents (#5/2024, #25/2024, and #33/2024). Viral genomes were also detected in fecal samples from rodents in the potential acute phase (#5/2024) and potential persistent phase (#11/2024, #20/2024, and #33/2024). Notably, infectious virus recovery from fecal specimens was limited to RNA-positive samples from individuals #5/2024 and #20/2024. These findings indicate that viral shedding in saliva, urine, and feces contributes to orthohantavirus transmission within natural rodent populations; saliva represents the most frequent and likely predominant source of infectious virus.

## DISCUSSION

The genus *Orthohantavirus* comprises numerous orthohantavirus species and genotypes (3, 45), some of which cause human infections and pose significant public health threats. Although viral evolution is thought to be closely linked to host speciation, host-switching events likely occurred during orthohantavirus evolution (1). Recently, it has been revealed that some orthohantaviruses can infect multiple species of rodents (9, 10). However, each orthohantavirus species seems to have the preferred natural host species. Hantavirus infection among natural animal hosts is generally believed to occur through direct physical contact, such as biting or fighting involving exposure to virus-laden saliva, or by inhalation of infectious aerosols from contaminated excreta. Infected hosts typically develop chronic asymptomatic infections. Although several studies using experimentally infected rodents have been conducted to elucidate the mechanisms of orthohantavirus maintenance and transmission, data from naturally infected rodents remain limited. This study was conducted to analyze the persistence and transmission characteristics of HOKV, a genotype of *O. puumalaense*, in its natural rodent host, *M. rufocanus bedfordiae*, under natural infection conditions. Our findings confirmed that HOKV establishes persistent infection within its natural reservoir without inducing overt histopathological lesions in major organs, consistent with previous reports of orthohantavirus infection in their rodent hosts (15, 23, 25, 46).

We have been conducting a series of epidemiological studies on HOKV infection in the *M. rufocanus bedfordiae* population within the same isolated forest in Tobetsu, Hokkaido, Japan, for more than 30 years. HOKV-infected individuals have been identified in each survey (10, 29, 31). The results of four independent surveys conducted from 2022, 2023, 2024, and 2025 showed that the HOKV infection rate in the natural rodent population was 11.4% (5/44), 3.6% (2/56), 20.5% (8/39), and 13.3% (8/60), respectively, indicating long-term maintenance of HOKV within the same host population. In this study, 22 of 23 HOKV-positive individuals were *M. rufocanus bedfordiae*, whereas one was *A. speciosus*. This is the direct evidence of HOKV infection in *A. speciosus*, although only a few seropositive *A. speciosus* were detected in the past. Therefore, this infection is likely the result of spillover from *M. rufocanus bedfordiae* (10). No antibodies or viral RNA were detected in *A. argenteus* individuals captured during these field surveys.

The dynamics of antibody responses and viral RNA prevalence in natural host populations are influenced by multiple ecological and host-related factors, making the characterization of orthohantavirus infection at the individual level particularly challenging (28, 47–49). To date, the infection status in natural reservoirs remains poorly understood, especially regarding the temporal progression of infection within individual animals. Although IgM is generally considered a sensitive marker of primary infection, its value to define the acute phase is limited because it may also be detected during viral reactivation or reinfection, as well as its prolonged persistence in some individuals (21, 24, 50). In rodent hosts infected with orthohantaviruses, the presence of IgM antibodies in both acute and persistent phases is not uncommon, potentially due to continuous antigenic stimulation by persisting viral antigens (24, 51). In this context, IgG avidity assays provide a valuable complementary approach for estimating the timing of infection. IgG avidity, defined as the binding strength between IgG antibodies and their corresponding antigens, increases progressively over the course of infection. Low-avidity IgG is typically indicative of recent infection, whereas high-avidity IgG is associated with past or persistent infection. This approach has been widely applied in both human and animal studies to discriminate between recent and chronic infections for several pathogens, including SEOV, PUUV, rubella virus, cytomegalovirus, *Toxoplasma gondii*, and *Neospora caninum* (42, 43, 51–54). Therefore, incorporating IgG avidity measurements alongside conventional serological assays may enhance the resolution of epidemiological analyses in orthohantavirus research.

In the present study, we analyzed IgM, IgG, neutralizing antibody responses, IgG avidity, and viral RNA in HOKV-infected natural hosts. The results suggested the presence of two phases of infection based on serological and molecular findings. Eighteen rodents exhibited concurrent viral RNA detection and high levels of IgG antibodies with strong neutralizing activity and high IgG avidity (≥64), suggesting that they are in a persistent infection state. In contrast, five individuals were considered to be in the acute phase of infection, characterized by detectable viral RNA together with low antibody titers and low IgG avidity (≤26). These findings may support the concept that orthohantaviruses can replicate within the hosts in either acute or persistent infection, regardless of specific antibody presence (21, 24, 29).

Quantitative analysis of viral load demonstrated widespread presence of HOKV RNA across multiple organs and in blood clots, with the highest copy numbers detected in the lungs, kidneys, and spleen. Consistent with these findings, IHC analysis revealed HOKV antigen signals in the same organs, most frequently in the lungs, kidneys, and spleen. The consistent detection pattern across potential acute and persistent phases of infection suggests that HOKV is maintained in multiple organs throughout the course of infection, which may be crucial for understanding the mechanisms of viral persistence. TEM further confirmed the presence of orthohantavirus-like particles in naturally infected rodents. Despite this systemic distribution, histopathological examination revealed no obvious lesions directly attributable to HOKV infection. Mild inflammatory changes were observed in the lungs of some infected individuals, but similar findings were also recorded in uninfected rodents. Previous studies of experimental infection with other orthohantaviruses, such as ANDV, PUUV, and SEOV, have identified distinct histological lesions in the lungs and/or kidneys (15, 22, 55). In natural settings, however, orthohantaviruses appear to cause limited pathological effects in their host species. Our observations in *M. rufocanus bedfordiae* during the potential acute phase of infection revealed only slight pathological alterations, although the influence of other pathogens cannot be excluded. Therefore, suitable experimental animal models are needed that accurately mimic the natural infection of orthohantaviruses in their hosts.

It is widely accepted that orthohantavirus infection in animal hosts is lifelong and asymptomatic. A more detailed analysis of orthohantavirus cellular tropism in wild rodents is essential to understand the mechanisms of viral persistence and dissemination within host organisms. Our findings suggested that HOKV antigens were predominantly localized with podoplanin-positive type I alveolar epithelial cells during the potential acute phase, whereas the virus antigen signals were more frequently associated with CD34-positive microvascular endothelial cells throughout the course of persistent infection. Previous studies of both natural and experimental infection with ANDV in *O. longicaudatus* and experimental infection with PUUV in *M. glareolus* revealed the localization of viral antigens in epithelial cells lining the alveoli and in endothelial cells of the septal capillaries (22, 25, 56, 57). These observations support the hypothesis that orthohantavirus infection in the respiratory epithelium may play a crucial role in the early phase of infection and may serve as a site of viral replication and release through the respiratory tract and/or salivary pathways (57–59). The infection pattern of orthohantaviruses is similar to that of other viruses, such as measles virus (60) and Epstein–Barr virus (61), which initially but transiently infect the respiratory epithelium to facilitate access to other cell types that serve as the primary sites of viral replication and dissemination. Furthermore, both Old World and New World orthohantaviruses have been shown to readily infect endothelial cells within various organs of animal hosts (15, 55, 62, 63). In humans, orthohantavirus infection of endothelial cells leads to increased vascular permeability, resulting in renal and pulmonary failure characteristic of HFRS and HCPS, respectively (64–66). In contrast, although viral antigens are widely distributed within the endothelium of organs in wild animal hosts, no major histopathological alterations have been observed. Despite extensive research, the cellular and molecular mechanisms underlying orthohantavirus persistence and pathogenesis remain poorly understood.

HOKV transmission is known to occur mainly through horizontal transmission among HOKV-infected *M. rufocanus bedfordiae* (10, 37). However, the mechanisms of virus maintenance and transmission within rodent populations remain unclear due to the lack of effective tools to monitor infection status in natural hosts. In this study, both viral RNA and infectious virus were detected in oral swabs, urine, and feces, indicating multiple potential transmission routes for HOKV in wild rodent populations. Notably, infectious virus was present in oral swabs from all 8 HOKV-positive individuals captured in the 2024 field survey, with high levels of viral RNA, regardless of the phase of infection; viral antigens were also observed in the salivary glands. In contrast, recovery of infectious virus from fecal and urinary specimens was limited in the infected animals. These findings suggest that saliva may serve as a major source of viral spread through aerosols and physical contact, such as biting and licking (27, 67).

In conclusion, investigation of natural HOKV infection in *M. rufocanus bedfordiae* provided crucial insights into orthohantavirus persistence, organ tropism, and viral shedding patterns. These findings further underscore the importance of establishing experimental models that closely mimic natural infection routes to advance our understanding of the mechanisms of orthohantavirus persistent infection, pathogenesis, and transmission in reservoirs.

## Data Availability

The data supporting the findings of this study are openly available in this article and are available from the corresponding author upon request. The S, M, and L RNA segments of the Tobetsu_29/2022 strain are available in GenBank under accession numbers LC790460.1, LC790465.1, and LC790470.1, respectively. The S, M, and L segments of the Samara_94/CG/2005 strain are available under accession numbers AB433845.2, AB433852.2, and AB574184.1, respectively.

## SUPPLEMENTAL MATERIAL

Figure S1 jvi.00321-26-s0001.tif Presence of orthohantavirus antigen in various organs of wild *M. rufocanus bedfordiae*.

Figure S2 jvi.00321-26-s0002.tif Orthohantavirus cell tropism in the lung of wild rodents.

Figure S3 jvi.00321-26-s0003.tif Orthohantavirus cell tropism in the kidney of wild rodents.

Supplemental legends jvi.00321-26-s0004.docx Descriptive legends for Fig. S1 to S3.

Table S1 jvi.00321-26-s0005.docx Virus loads in organs and biological samples from naturally infected wild rodents.

ASM does not own the copyrights to Supplemental Material that may be linked to, or accessed through, an article. The authors have granted ASM a non-exclusive, world-wide license to publish the Supplemental Material files. Please contact the corresponding author directly for reuse.
