# Supplementary figures and images for: Maintenance of Hokkaido virus, a genotype of Orthohantavirus puumalaense, in the rodent host Myodes rufocanus bedfordiae under natural conditions
Source: J Virol. 2026 Jun 30;100(7):e00321-26. doi: 10.1128/jvi.00321-26 (PMC13386903; doi:10.1128/jvi.00321-26)

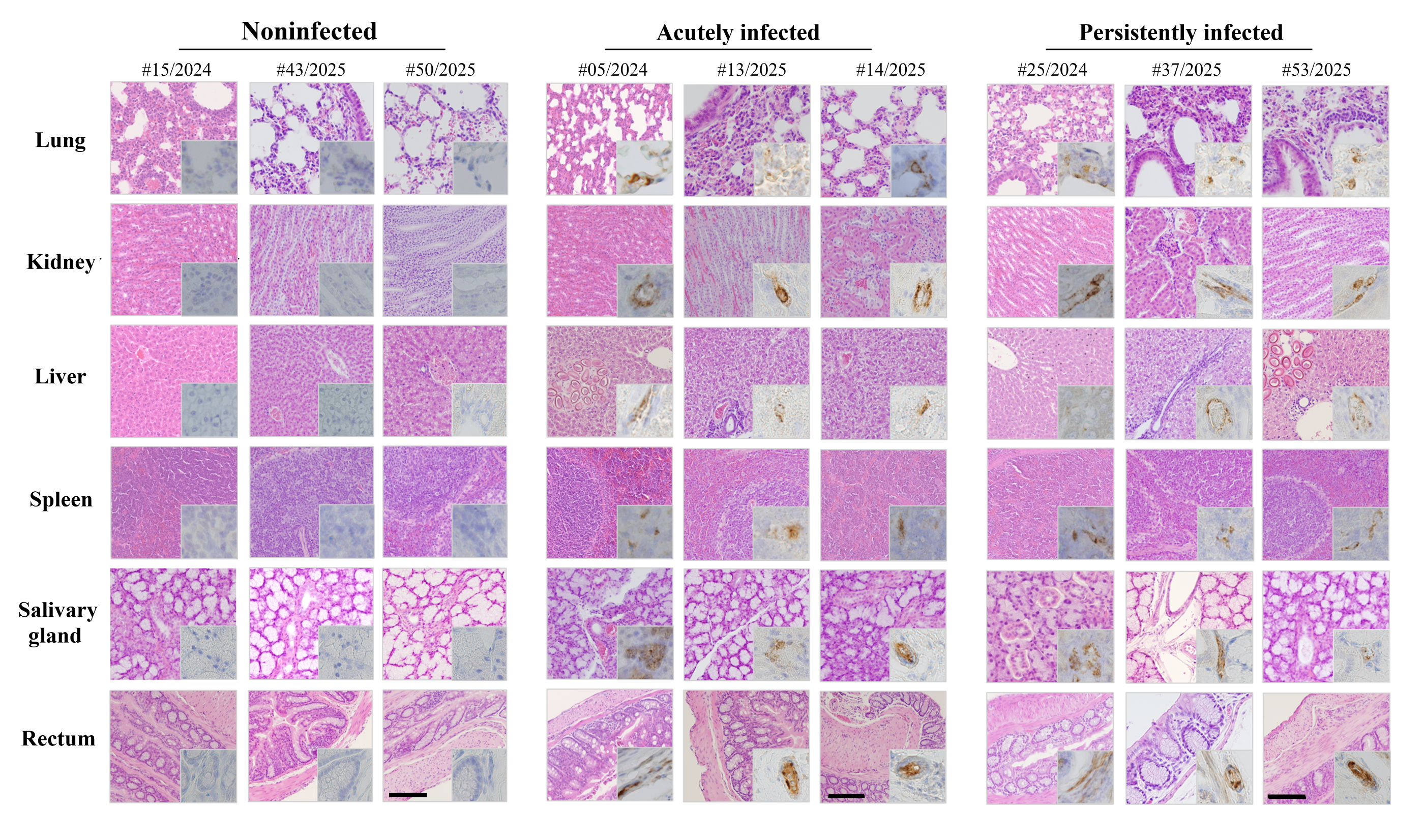

Supplement: Figure S1 — Presence of orthohantavirus antigen in various organs of wild M. rufocanus bedfordiae. [file jvi.00321-26-s0001.tif]

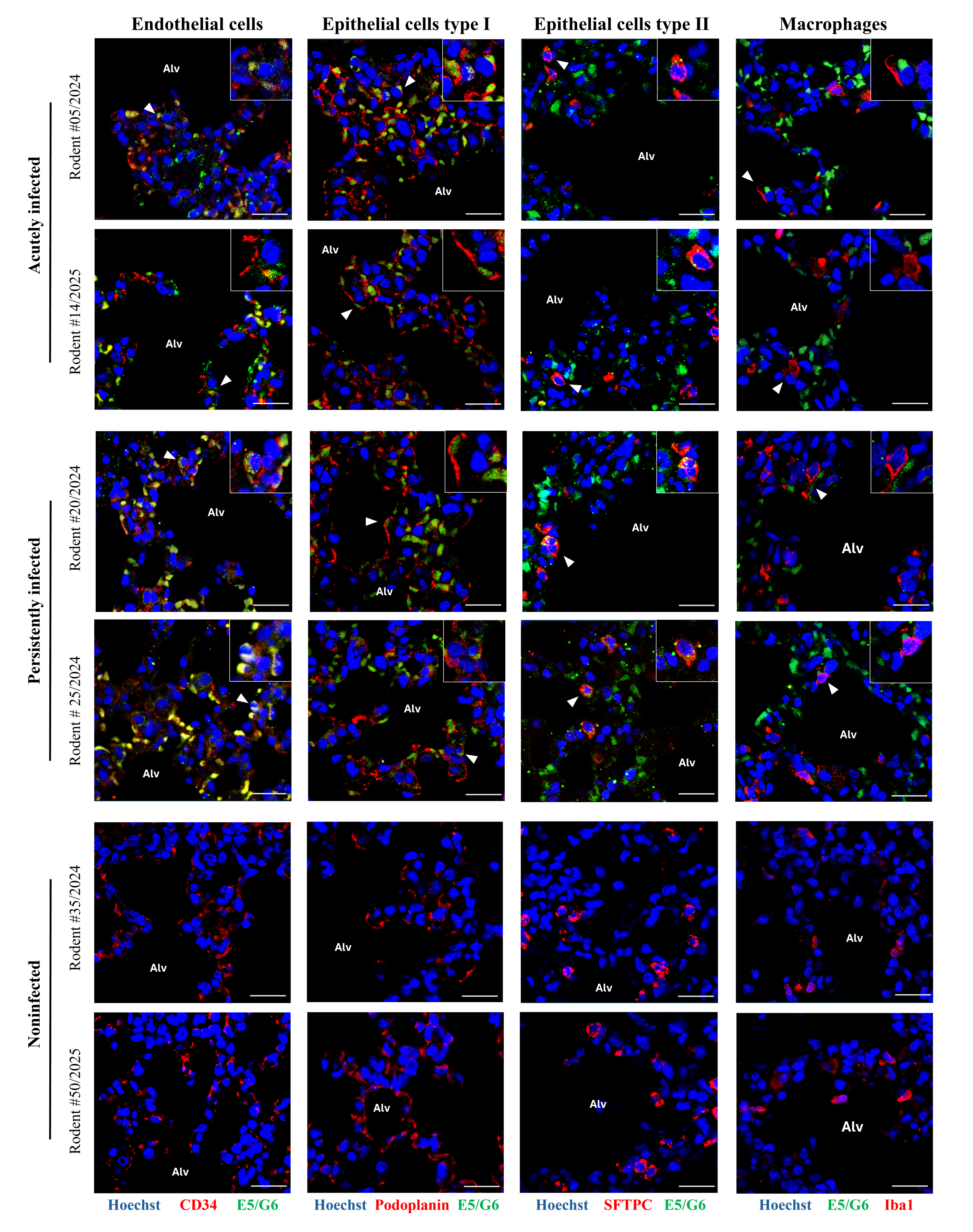

Supplement: Figure S2 — Orthohantavirus cell tropism in the lung of wild rodents. [file jvi.00321-26-s0002.tif]

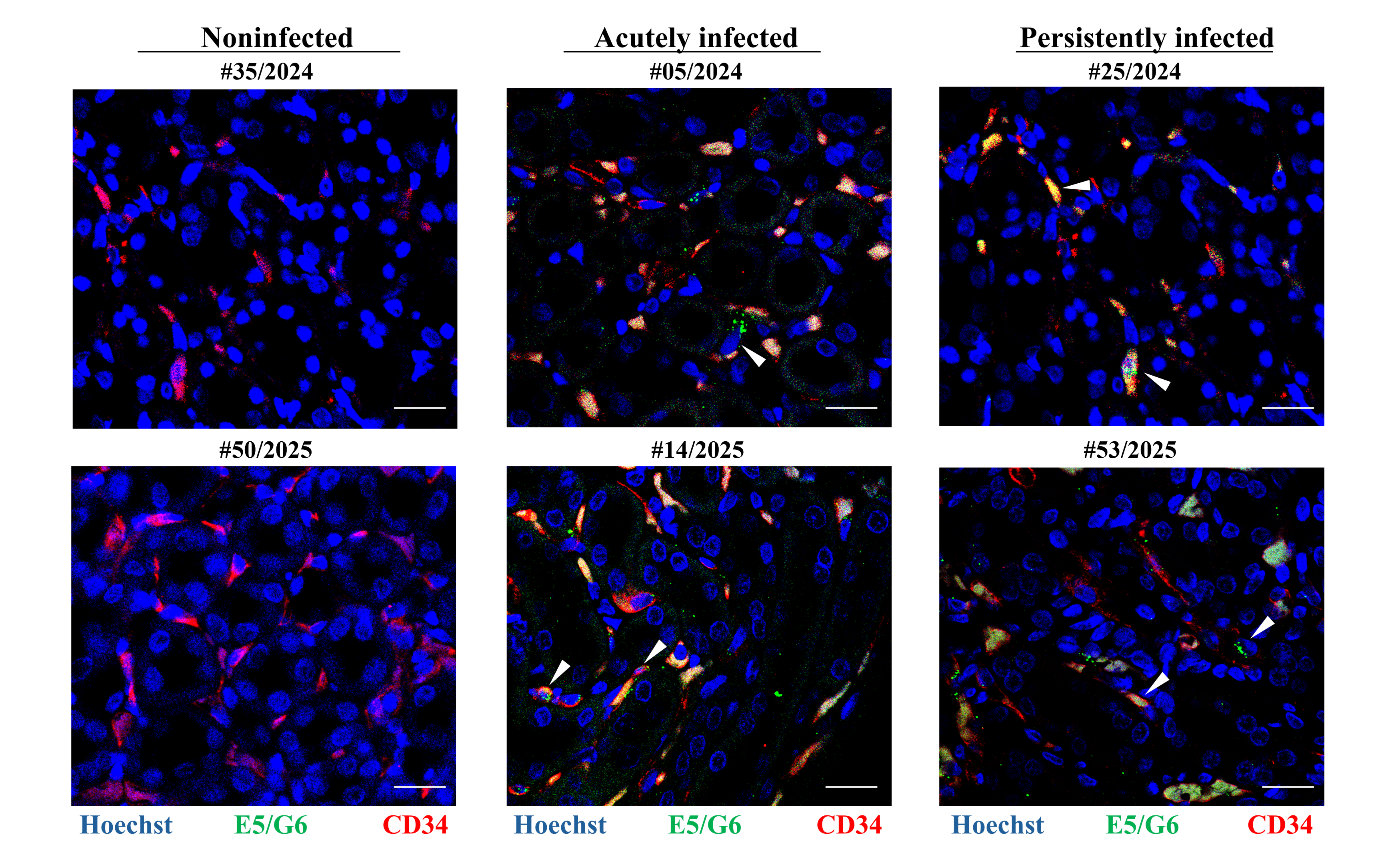

Supplement: Figure S3 — Orthohantavirus cell tropism in the kidney of wild rodents. [file jvi.00321-26-s0003.tif]
