# Supplementary material for: Maintenance of Hokkaido virus, a genotype of Orthohantavirus puumalaense, in the rodent host Myodes rufocanus bedfordiae under natural conditions
Source: J Virol. 2026 Jun 30;100(7):e00321-26. doi: 10.1128/jvi.00321-26 (PMC13386903; doi:10.1128/jvi.00321-26)
Supplement: Supplemental legends — Descriptive legends for Fig. S1 to S3. [file jvi.00321-26-s0004.docx]

**Supplementary Fig S1.** Presence of orthohantavirus antigen in various organs of wild *M. rufocanus bedfordiae*. Pathological and immunohistochemical examination of lung, kidney, liver, spleen, salivary gland, and rectum of naturally infected rodents. Representative sections are shown for organs of three animals per group, including noninfection (#15/2024, #43/2025, and #50/2025), potential acute infection (#05/2024, #13/2025, and #14/2025), and potential persistent infection (#25/2024, #37/2025, and #53/2025). Sections were stained with haematoxylin & eosin (H&E; scale bars, 200 µm), or by immunohistochemistry for orthohantavirus N (inset, 100 µm).

**Supplementary Fig S2.** Orthohantavirus cell tropism in the lung of wild rodents. Two representative immunofluorescence images per group are shown for lung sections from *M. rufocanus bedfordiae* with potential acute infection (#05/2024 and #14/2025), potential persistent infection (#20/2024 and #25/2024), and noninfection (#35/2024 and #50/2025). Sections were stained with antibodies against orthohantavirus antigen and different cell type markers. Orthohantavirus N was detected by monoclonal antibody E5/G6 (green). Microvascular endothelial cells, type I alveolar epithelial cells, type II alveolar epithelial cells, and macrophages were determined by cellular markers of CD34, podoplanin, SFTPC, and Iba-1, respectively (red). The nuclei were visualized using Hoechst 33342 staining (blue). Top right inset images offer a zoom-in of individually infected cells as indicated by arrowheads. Alveolar lumen (Alv). Scale bars, 20 μm.

**Supplementary Fig S3.** Orthohantavirus cell tropism in the kidney of wild rodents. Representative images of kidney sections from *M. rufocanus bedfordiae,* showing two representative animals per group, including potential acute infection (#05/2024 and #14/2025), potential persistent infection (#25/2024 and #53/2025), and noninfection (#35/2024 and #50/2025). Immunofluorescence staining with antibodies for orthohantavirus antigen (green) and microvascular endothelial cells, CD34 (red). The nuclei were visualized using Hoechst 33342 staining (blue). Representative of the co-localization of endothelial cell signals and viral antigen signals as indicated by white arrow heads. Scale bars, 20 μm.
