# Supplementary material for: Maintenance of Hokkaido virus, a genotype of Orthohantavirus puumalaense, in the rodent host Myodes rufocanus bedfordiae under natural conditions
Source: J Virol. 2026 Jun 30;100(7):e00321-26. doi: 10.1128/jvi.00321-26 (PMC13386903; doi:10.1128/jvi.00321-26)
Supplement: Table S1 — Virus loads in organs and biological samples from naturally infected wild rodents. [file jvi.00321-26-s0005.docx]

**Supplementary Table 1. Virus loads in organs and biological samples from naturally infected wild rodents**

| Rodent No.  /survey year | Infection phase | Viral load (Real-time PCR: viral RNA copies/ µg RNA) | | | | | | | | | | |
| --- | --- | --- | --- | --- | --- | --- | --- | --- | --- | --- | --- | --- |
|  |  | Lung | Kidney | Liver | Spleen | Heart | Blood clot | Salivary gland | Oral swabs | Rectum | Feces | Urine |
| #4/2022 | Acute | 4.0×10^4^ | 5.9×10^3^ | 4.2×10^2^ | 1.5×10^5^ | NA | 4.7×10^3^ | NA | NA | NA | NA | NA |
| #5/2024 | Acute | 1.6×10^7^ | 3.4×10^6^ | 5.6×10^4^ | 6.6×10^6^ | 1.2×10^4^ | 6.3×10^4^ | 3.3×10^2^ | 8.0×10^4^ | 2.5×10^6^ | 6.4×10^1^ | ND |
| #28/2024 | Acute | 4.9×10^5^ | 2.3×10^6^ | 5.8×10^4^ | 5.2×10^5^ | 5.1×10^4^ | 2.4×10^4^ | 1.2×10^3^ | 4.9×10^4^ | 1.5×10^4^ | NA | NA |
| #13/2025 | Acute | 7.1×10^6^ | 2.8×10^5^ | 4.3×10^3^ | 2.6×10^5^ | 2.1×10^4^ | 5.7×10^4^ | 5.2×10^3^ | NA | 7.1×10^4^ | NA | NA |
| #14/2025 | Acute | 2.9×10^6^ | 2.4×10^4^ | 3.6×10^3^ | 3.1×10^4^ | 1.2×10^4^ | 1.4×10^4^ | 4.6×10^1^ | NA | 9.1×10^1^ | NA | NA |
| #9/2022 | Persistent | 6.1×10^3^ | 1.6×10^1^ | 5.3×10^2^ | 2.3×10^3^ | NA | 1.2×10^2^ | NA | NA | NA | NA | NA |
| #25/2022 | Persistent | 1.7×10^4^ | 4.9×10^3^ | 8.0×10^2^ | 1.8×10^4^ | NA | 2.2×10^4^ | NA | NA | NA | NA | NA |
| #29/2022 | Persistent | 6.9×10^5^ | 3.3×10^4^ | 9.5×10^3^ | 9.4×10^4^ | NA | 4.6×10^3^ | NA | NA | NA | NA | NA |
| #38/2022 | Persistent | 1.3×10^3^ | 2.2×10^2^ | 8.7×10^3^ | 6.9×10^2^ | NA | 6.5×10^2^ | NA | NA | NA | NA | NA |
| #37/2023 | Persistent | 2.4×10^4^ | 2.2×10^3^ | 9.0×10^2^ | 5.3×10^4^ | NA | 3.1×10^3^ | NA | NA | NA | NA | NA |
| #48/2023 | Persistent | 1.7×10^4^ | 3.3×10^4^ | 1.5×10^2^ | 5.3×10^3^ | NA | 4.3×10^3^ | NA | NA | NA | NA | NA |
| #11/2024 | Persistent | 2.7×10^7^ | 5.5×10^5^ | 8.1×10^4^ | 6.8×10^6^ | 5.4×10^4^ | 1.1×10^4^ | ND | 3.0×10^4^ | 3.9×10^5^ | 1.5×10^1^ | 9.5×10^4^ |
| #16/2024 | Persistent | 1.3×10^6^ | 8.3×10^4^ | 4.2×10^3^ | 7.3×10^5^ | 2.8×10^4^ | 6.6×10^3^ | ND | 4.6×10^4^ | ND | ND | NA |
| #20/2024 | Persistent | 2.1×10^7^ | 3.4×10^6^ | 8.1×10^5^ | 6.2×10^6^ | 5.7×10^4^ | 1.0×10^4^ | 8.9×10^3^ | 2.5×10^4^ | 3.6×10^4^ | 1.1×10^1^ | 5.0×10^3^ |
| #25/2024 | Persistent | 8.3×10^4^ | 1.9×10^4^ | 4.3×10^3^ | 5.3×10^5^ | 9.7×10^3^ | 3.8×10^2^ | 1.2×10^2^ | 1.4×10^3^ | ND | ND | ND |
| #27/2024 | Persistent | 4.1×10^6^ | 2.2×10^6^ | 6.9×10^3^ | 4.7×10^5^ | 1.1×10^5^ | 2.3×10^4^ | 2.1×10^3^ | 1.1×10^5^ | 1.5×10^4^ | NA | 2.4×10^4^ |
| #33/2024 | Persistent | 6.8×10^5^ | 1.1×10^4^ | 1.2×10^3^ | 2.8×10^4^ | 5.6×10^3^ | 5.7×10^3^ | ND | 3.1×10^3^ | 4.5×10^4^ | 1.8×10^1^ | ND |
| #18/2025 | Persistent | 2.9×10^7^ | 5.0×10^5^ | 4.7×10^4^ | 3.8×10^6^ | 4.0×10^4^ | 1.1×10^4^ | ND | NA | 8.0×10^4^ | NA | NA |
| #24/2025 | Persistent | 2.7×10^5^ | 1.3×10^6^ | 8.1×10^3^ | 4.8×10^4^ | 1.1×10^4^ | 8.6×10^3^ | 5.3×10^2^ | NA | 8.1×10^3^ | NA | NA |
| #37/2025 | Persistent | 5.2×10^6^ | 5.6×10^5^ | 3.4×10^3^ | 6.4×10^5^ | 2.1×10^3^ | 1.3×10^4^ | 1.8×10^3^ | NA | 1.6×10^3^ | NA | NA |
| #53/2025 | Persistent | 1.7×10^5^ | 8.3×10^5^ | 2.6×10^2^ | 2.9×10^3^ | 1.0×10^4^ | 2.4×10^3^ | 4.1×10^4^ | NA | 5.0×10^5^ | NA | NA |
| #58/2025 | Persistent | 4.6×10^5^ | 2.4×10^4^ | 2.9×10^3^ | 5.2×10^4^ | 3.1×10^3^ | 1.8×10^3^ | 3.5×10^2^ | NA | 3.6×10^4^ | NA | NA |
| #59/2025 | Persistent | 5.2×10^5^ | 1.6×10^6^ | 6.9×10^3^ | 3.1×10^4^ | 2.2×10^3^ | 8.4×10^3^ | 4.0×10^4^ | NA | 3.1×10^4^ | NA | NA |
| #15/2024 | Noninfected | ND | ND | ND | ND | ND | ND | ND | ND | ND | ND | ND |
| #24/2024 | Noninfected | ND | ND | ND | ND | ND | ND | ND | ND | ND | ND | ND |
| #35/2024 | Noninfected | ND | ND | ND | ND | ND | ND | ND | ND | ND | ND | ND |
| #43/2025 | Noninfected | ND | ND | ND | ND | ND | ND | ND | NA | ND | NA | NA |
| #50/2025 | Noninfected | ND | ND | ND | ND | ND | ND | ND | NA | ND | NA | NA |

ND, not detected; NA, not available.
